# Mechanical impact of regional structural deterioration and tissue-level compensation on proximal femur trabecular bone

**DOI:** 10.3389/fbioe.2024.1448708

**Published:** 2024-09-09

**Authors:** Chenglong Feng, Ke Zhang, Shi Zhan, Yuxiong Gan, Xinhao Xiang, Wenxin Niu

**Affiliations:** ^1^ Shanghai Yangzhi Rehabilitation Hospital (Shanghai Sunshine Rehabilitation Center), School of Medicine, Tongji University, Shanghai, China; ^2^ Laboratory of Biomechanics and Rehabilitation Engineering, School of Medicine, Tongji University, Shanghai, China; ^3^ Biomechanical Laboratory of Orthopedic Surgery Department, Shanghai Jiao Tong University Affiliated Sixth People’s Hospital, Shanghai, China

**Keywords:** osteoporosis, trabecular bone mechanics, proximal femur, structural deterioration, tissue-level mechanical property

## Abstract

**Introduction:**

Osteoporosis-induced changes in bone structure and composition significantly reduce bone strength, particularly in the human proximal femur. This study examines how these changes affect the mechanical performance of trabecular bone to enhance diagnosis, prevention, and treatment strategies.

**Methods:**

A proximal femur sample was scanned using micro-CT at 40 μm resolution. Five regions of interest were selected within the femoral head, femoral neck, and greater trochanter. Structural models simulating various stages of osteoporosis were created using image processing software. Micro-finite element analysis evaluated the mechanical properties of trabecular bone under different conditions of structural deterioration and tissue-level elastic modulus variations. The combined effects of structural deterioration and tissue-level mechanical properties on trabecular bone mechanical performance were further analyzed.

**Results:**

The mechanical performance of trabecular bone generally follows a power-law relationship with its microstructural characteristics. However, in any specific region, the apparent mechanical properties linearly decrease with structural deterioration. The femoral neck and greater trochanter are more sensitive to structural deterioration than the femoral head. A 5% bone mass loss in the femoral head led to a 7% reduction in mechanical performance, while the femoral neck experienced a 12% loss. Increasing tissue-level elastic modulus improved mechanical performance, partially offsetting bone mass reduction effects.

**Conclusion:**

Trabecular bone in low bone mass regions is more affected by bone mass loss. Structural deterioration primarily reduces bone strength, but improvements in tissue-level properties can mitigate this effect, especially in early osteoporosis. Targeted assessments and interventions are crucial for effective management. Future research should explore heterogeneous deterioration models to better understand osteoporosis progression.

## 1 Introduction

Osteoporosis is a common skeletal disease characterized by reduced bone mass and deteriorating bone quality, leading to increased bone fragility and a higher risk of fractures (Black and Rosen, 2016; Anam and Insogna, 2021). Trabecular bone, an essential load-bearing component of the skeleton, plays a crucial role in the mechanical performance of the bone structure (Nazarian and Christiansen, 2015). Due to its porous structure and high metabolic activity, trabecular bone undergoes more significant structural and compositional changes due to osteoporosis compared to cortical bone (McCreadie et al., 2006). Understanding the osteoporosis-induced structural and compositional changes in trabecular bone, as well as their impact on the overall mechanical performance of bone structures, is essential for improving the diagnosis and treatment of osteoporosis.

Structural deterioration caused by osteoporosis is a key factor in the reduced mechanical performance of trabecular bone (Mittra et al., 2005). Based on two population-based cohorts, Chapurlat et al. (Chapurlat et al., 2020) conducted a large-scale study with over 2,000 subjects and found significant structural deterioration in osteoporotic patients, which was closely associated with an increased risk of fractures. Studies have demonstrated that osteoporosis-induced changes in bone mass, microstructural characteristics, and mechanical properties are highly interrelated (McCalden et al., 1997; Silva and Gibson, 1997). Microstructural changes, such as reductions in trabecular number, thickness, and connectivity, and a shift from plate-like to rod-like trabeculae, weaken the load-bearing capacity of trabecular bone (Liu et al., 2006; Wang et al., 2015a; Du et al., 2019). These changes are considered the primary reason for the high fracture risk in osteoporotic patients. Therefore, investigating the impact of osteoporosis-induced structural changes on the mechanical performance of trabecular bone is crucial for the diagnosis and prevention of osteoporosis-related fractures.

In addition to structural deterioration, changes in bone tissue composition due to osteoporosis also affect the mechanical performance of trabecular bone. Studies have shown that osteoporotic bone tissue exhibits significantly reduced mineral content, lower degrees of mineralization, and decreased mineral-to-matrix ratios compared to healthy bone tissue, resulting in diminished mechanical performance at the tissue level (Boskey et al., 2005; Frank et al., 2021; Paschalis et al., 2021). Moreover, the uneven mineralization of bone tissue further compromises the mechanical integrity of trabecular bone (Busse et al., 2009; Zhao et al., 2020). However, O’Sullivan et al. (2020) have pointed out that bone mineralization following initial bone loss in osteoporosis can reinforce the trabecular network, compensating for bone loss and increased loading. Although changes in the material-level mechanical properties of bone tissue are recognized to influence overall mechanical performance, the extent of this impact remains unclear, particularly whether tissue-level hardening can compensate for the negative effects of bone loss on structural mechanical performance.

The progression of osteoporosis is a gradual process, and the changes in bone mechanical performance resulting from structural and material alterations need to be systematically analyzed. While osteoporotic animal models provide some insights, accurately tracking these changes over time remains challenging (Yousefzadeh et al., 2020). Previous studies have often used periodic tracking or age-group comparisons to explore patterns of structural deterioration and trabecular composition changes (McGivern et al., 2020; Yokota et al., 2020; Xi et al., 2021). However, these methods often fail to capture progressive changes accurately (Guha et al., 2020; Pumberger et al., 2020).

Micro-finite element analysis (μFEA) offers a solution for quantitatively studying the combined effects of structural and tissue-level properties on the mechanical performance of trabecular bone. This technique can accurately simulate the mechanical behavior of trabecular bone microstructures (Gong et al., 2016; Yu et al., 2020; Zhan et al., 2024). Through image processing and modeling, μFEA allows for a quantitative analysis of how structural deterioration affects bone mechanical performance. Additionally, it enables the adjustment of mechanical properties in bone tissues with varying structures, facilitating the study of tissue-level mechanical properties in the context of osteoporosis-induced mechanical decline (Liu et al., 2019; Li et al., 2021). Huang et al. (2023) further validated the applicability of this method in osteoporosis research by combining it with 3D printing technology. However, these studies primarily focus on mouse bone tissues, which differ significantly in microstructure from human bones. Consequently, the relevance of these findings to human bone remains uncertain, and further investigation is needed to understand the influence of tissue-level mechanical properties on overall mechanical performance.

This study aims to quantitatively assess the impact of structural deterioration and tissue-level mechanical changes on the apparent mechanical performance of trabecular bone. Given the high risk of fragility fractures and regional microstructural differences in the proximal femur, our research focuses on the changes occurring in various parts of the proximal femur during osteoporosis. By simulating the structural deterioration process of trabecular bone and precisely controlling variables such as structural and tissue-level mechanical properties, we systematically analyze the relationship between these factors and trabecular bone mechanical performance. This study seeks to provide new insights for the prevention, diagnosis, and treatment of osteoporosis and fragility fractures.

## 2 Materials and methods

### 2.1 Micro-CT scanning and ROI selection

**FIGURE 1:**
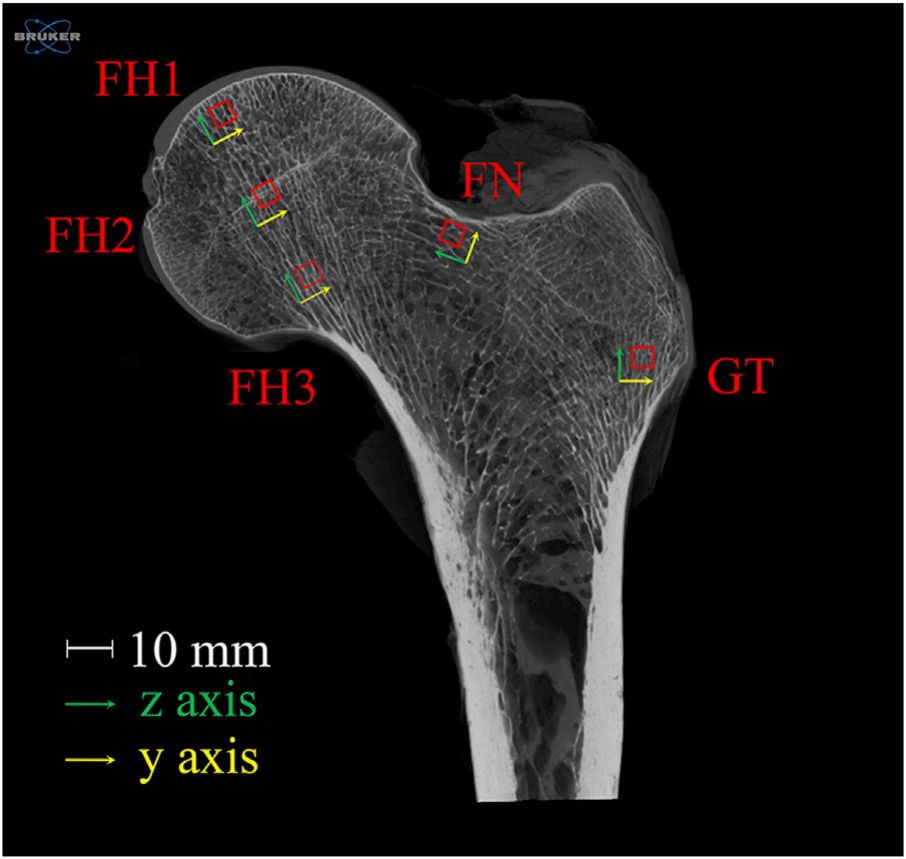
Selection and orientation of ROIs in the proximal femur. The plane shown is the section along the femoral neck axis; FH2 is located at the center of the femoral head. FH1, FH2, and FH3 are equidistant and collinear along the *z*-axes, with FH1 located 2 mm from the subchondral bone plate. FN is positioned 2 mm below the upper edge of the femoral neck, and GT is 4 mm from the lateral point of the greater trochanter. FH stands for femoral head, FN for femoral neck, and GT for greater trochanter.

A healthy proximal femur sample, extending from the femoral head to the greater and lesser trochanters, was obtained from a 43-year-old male donor. The donor had no history of lower limb fractures, bone tumors, osteoporosis, osteoarthritis, or any other metabolic bone diseases. The sample was scanned using a Skyscan 1273 Micro-CT (Bruker, United States) at 40 μm resolution. Five cubic regions of interest (ROIs) with 4 mm side lengths were selected from the upper, central, and lower parts of the femoral head (FH), upper femoral neck (FN), and greater trochanter (GT). The *x*-axis of each ROI was oriented anteriorly, and the *z*-axis was aligned with the trabecular orientation. The positions and orientations of the ROIs are shown in Figure 1. The plane shown is the section along the femoral neck axis; FH2 is located at the center of the femoral head. FH1, FH2, and FH3 are equidistant and collinear along the *z*-axes, with FH1 located 2 mm from the subchondral bone plate. FN is positioned 2 mm below the upper edge of the femoral neck, and GT is 4 mm from the lateral point of the greater trochanter.

It is important to note that the ROIs selected in this study are all located in the loading regions of the proximal femur. Specifically, FH1, FH2, and FH3 are in the principal compressive group, while FN and GT are in the principal tensile group. Bone structure exhibits functional adaptability, with trabecular orientation aligning with the principal stress trajectories (Boyle and Kim, 2011). These regions make significant contributions to the load-bearing function of the proximal femur. Therefore, investigating the relationship between structural changes and mechanical function in these regions is more representative.

### 2.2 Osteoporosis structural model establishment and microstructural analysis

**FIGURE 2:**
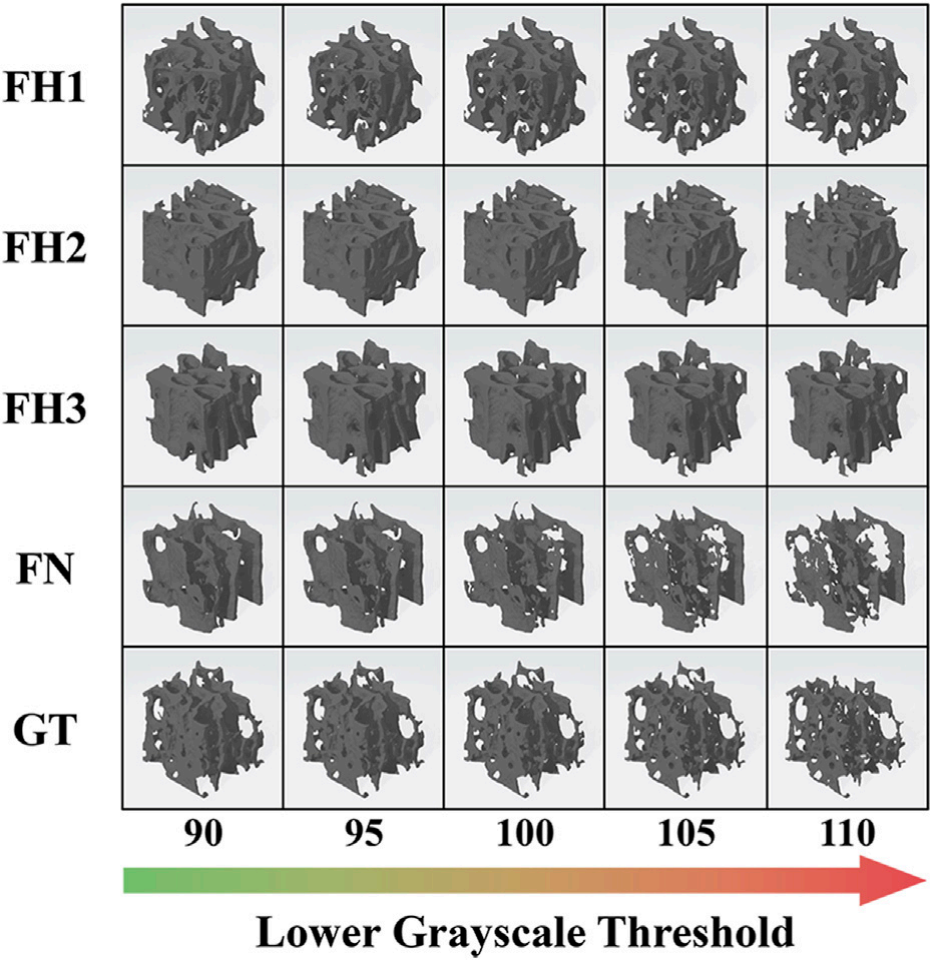
Structural models representing different stages of osteoporosis progression.

In this study, the original healthy bone structure in each ROI was restored using a lower grayscale threshold of 90. Severe structural deterioration can be simulated with a threshold of 110. Thus, structural models representing the progression of osteoporosis at five threshold levels (90, 95, 100, 105, and 110) were built using Simpleware software (Synopsys Inc., United States), as shown in Figure 2. Microstructural parameters, such as bone volume fraction (BV/TV), structure model index (SMI), trabecular number (Tb.N), and trabecular thickness (Tb.Th), in different ROIs and deterioration stages were analyzed using CTAn software (Bruker, United States).

### 2.3 Micro-finite element analysis

**FIGURE 3:**
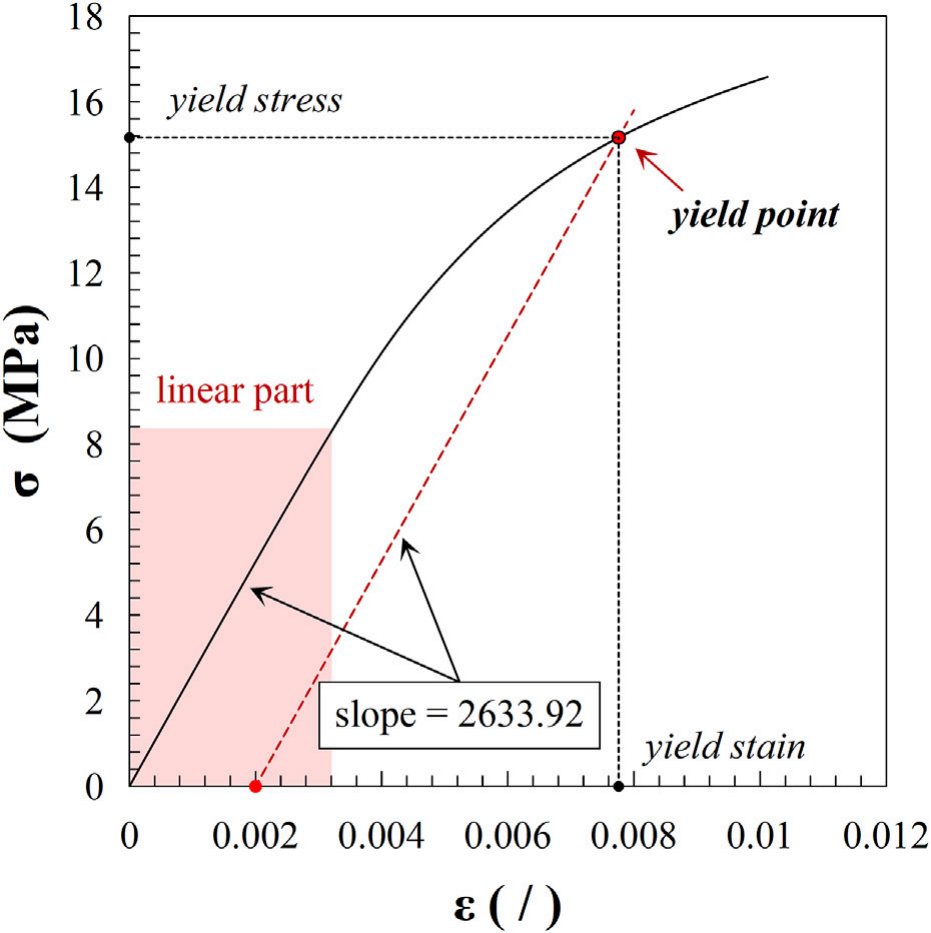
Stress-strain curve from FEA results of FH1 (lower grayscale threshold: 90; tissue-level elastic modulus: 18 GPa). The curve illustrates the linear deformation region, with the slope representing the apparent elastic modulus. The yield point is identified by the intersection with the 0.2% strain offset line, indicating the model’s apparent yield strain and apparent yield strength.

A validated μFEA method with over 95% accuracy was used to calculate the apparent mechanical properties of trabecular bones (Gong et al., 2016; Yu et al., 2020). In last section, we generated 25 three-dimensional models of cubic trabecular bone structures using Simpleware. Based on these models, we utilized the scanFE module in Simpleware to construct voxel mesh models for each structure, corresponding to the scanning resolution of the original images. The mesh models were saved in .inp format and subsequently imported into Abaqus 6.14 (Dassault Systemes, France) for material property assignment, loading, and simulation analysis. A 1% compressive strain was applied along the *z*-axis. According to previous studies, the tissue-level elastic modulus of trabecular material ranged from 6 to 27 GPa (Giambini et al., 2013; Yu et al., 2020). The tissue-level elastic modulus of healthy trabecular bones was set to 18 GPa in this study (Gong et al., 2016). Variations in tissue-level trabecular stiffness were simulated with elastic modulus of 10, 14, 22, and 26 GPa. The tensile and compressive yield points of trabecular material were set at 0.4% and 0.8% strain, respectively, with the post-yield modulus reduced to 5% of the elastic modulus. Apparent mechanical properties, including apparent elastic modulus (E_apparent_), apparent yield strain (ε_apparent_), and apparent yield strength (σ_apparent_), were derived from the stress-strain data. The apparent elastic modulus was defined as the slope of the elastic region of the stress-strain curve, and the yield point was determined using the 0.2% offset method. Figure 3 shows a typical stress-strain curve obtained from FEA, based on the simulation results of FH1 (with a lower grayscale threshold of 90 and a tissue-level elastic modulus of 18 GPa). The slope represents the linear deformation and corresponds to the apparent elastic modulus. The yield point is determined by the intersection of the stress-strain curve and a line drawn through the 0.2% strain offset with the same slope as the apparent elastic modulus. The coordinates of the yield point indicate the apparent yield strain and apparent yield strength of the model.

### 2.4 Statistical analysis

Statistical analyses were conducted using SPSS 20.0 (IBM Corp. United States). Spearman correlation analysis was utilized to assess the relationships between microstructural parameters and the apparent mechanical properties of trabecular bone. Linear and nonlinear regression analyses were performed to examine the relationships between apparent mechanical properties and bone mass, the rate of change in apparent mechanical properties and bone mass, and the impact of tissue-level mechanical properties on apparent mechanical properties. A bilateral *p*-value of less than 0.05 was considered statistically significant.

## 3 Results

### 3.1 Microstructural changes due to structural deterioration

**FIGURE 4:**
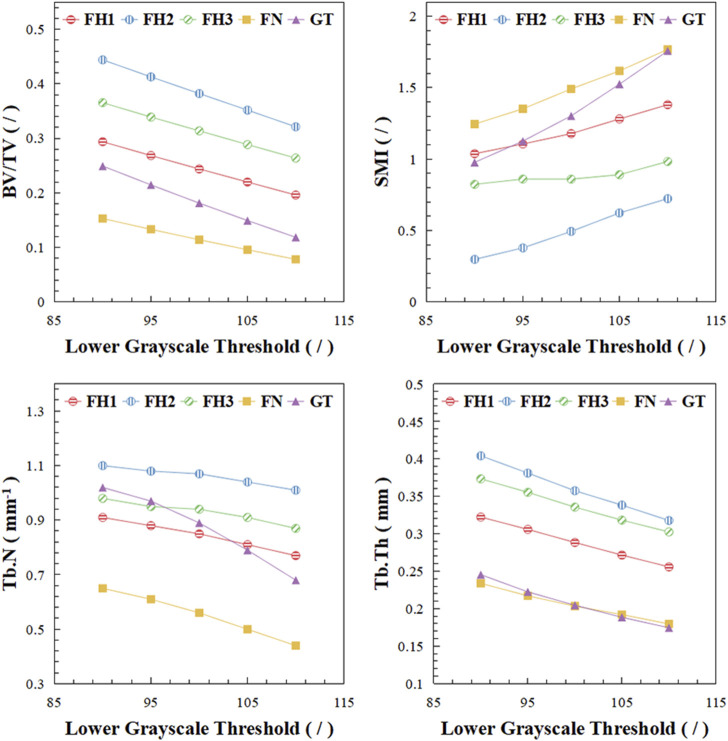
Microstructural changes of trabecular bone in different regions. BV/TV stands for bone volume fraction, SMI stands for structure model index, Tb.N stands for trabecular number, Tb.Th stands for trabecular thickness.

The microstructural characteristics of trabecular bone varied across different regions of the proximal femur, as shown in Figure 4. In Figure 4, BV/TV represents the bone volume fraction, indicating the proportion of bone tissue within a unit volume. SMI, or Structure Model Index, reflects the balance between rod-like and plate-like structures within a 3D object; an ideal plate has an SMI value of 0, while an ideal cylinder has an SMI value of 3. Tb.N denotes the trabecular number, and Tb.Th represents the trabecular thickness. The femoral head exhibited the highest bone mass, followed by the greater trochanter, with the lowest bone volume fraction observed in the femoral neck. This pattern was consistent with the distribution of trabecular thickness. As the lower grayscale threshold increased, trabecular bone volume, thickness, and number progressively decreased, with trabeculae transitioning from plate-like to rod-like structures, reflecting osteoporosis progression. The trabecular morphology and number (SMI, Tb.N) in the greater trochanter were more sensitive to changes in bone mass compared to other regions.

### 3.2 Relationship between structural deterioration and apparent mechanical properties

**FIGURE 5:**
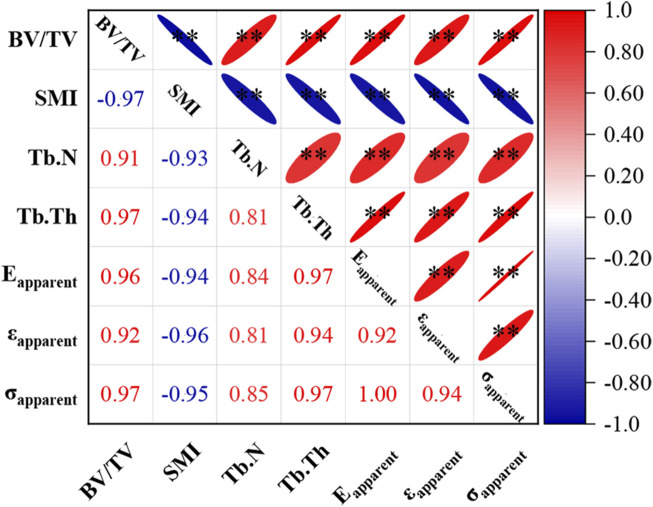
Correlation between microstructural parameters and apparent mechanical properties of trabecular bone. * indicates *p* < 0.05, ** indicates *p* < 0.01. E_apparent_ stands for apparent elastic modulus, ε_apparent_ stands for apparent yield strain, σ_apparent_ stands for apparent yield strength.

Correlation analysis revealed significant relationships between various microstructural parameters and the apparent mechanical properties of trabecular bone (Figure 5). Except for the structure model index, all other parameters exhibited significant positive correlations. The correlation between trabecular number and apparent mechanical performance was relatively weaker (|r| values between 0.81 and 0.85). In contrast, correlation coefficients for the remaining microstructural parameters with apparent mechanical performance had absolute values exceeding 0.9.

**FIGURE 6:**
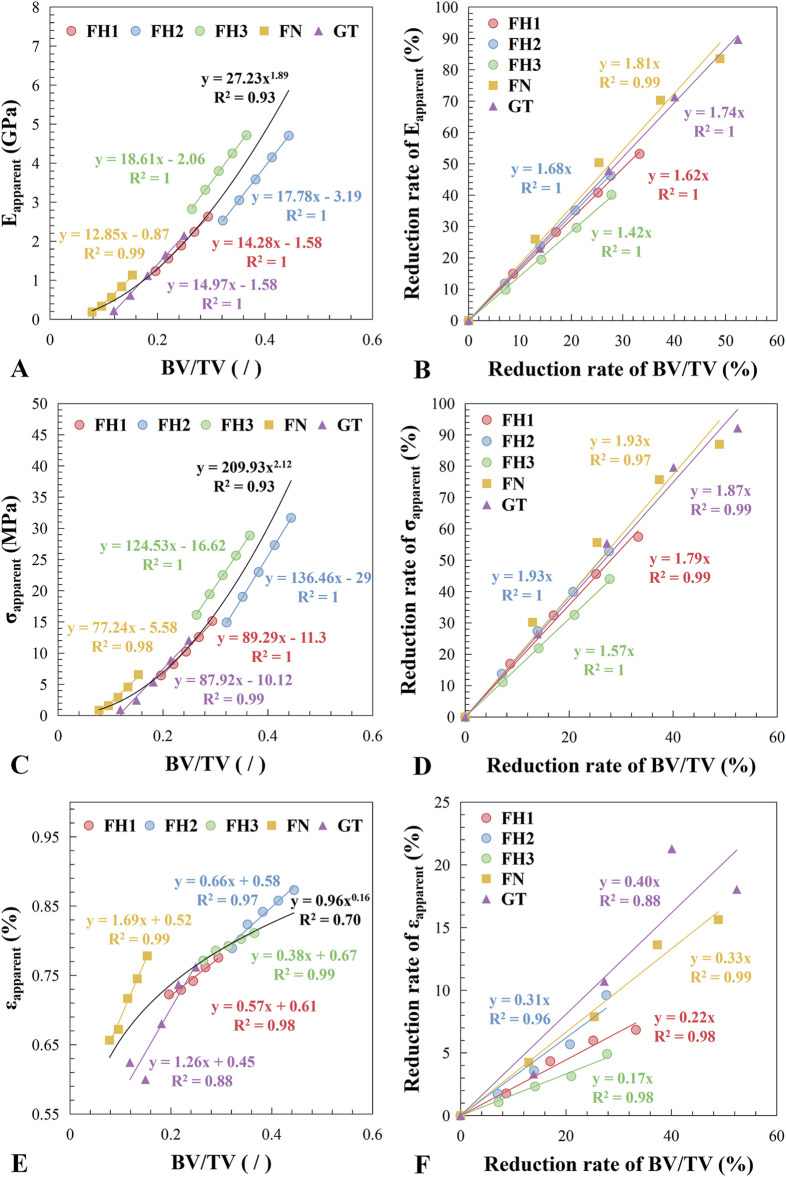
Relationship between apparent mechanical properties and bone loss. **(A)** Apparent elastic modulus vs. bone volume fraction. **(B)** Reduction rate of apparent elastic modulus vs. reduction rate of bone volume fraction. **(C)** Apparent yield strength vs. bone volume fraction. **(D)** Reduction rate of apparent yield strength vs. reduction rate of bone volume fraction. **(E)** Apparent yield strain vs. bone volume fraction. **(F)** Reduction rate of apparent yield strain vs. reduction rate of bone volume fraction. The fitting results for each region are indicated in the figure by the legend colors, while the overall fitting results covering all regions are presented in black.

As shown in Figure 6A, the overall relationship between the apparent elastic modulus and bone mass (BV/TV) follows an approximate power-law distribution (R^2^ = 0.93). However, in different regions of the proximal femur, the apparent elastic modulus exhibits a clear linear decrease with decreasing bone mass (R^2^ ≥ 0.99), with varying slopes. Specifically, regions with higher bone mass, such as the central (FH2) and lower (FH3) parts of the femoral head, have steeper slopes of 17.78 and 18.61, respectively. In contrast, regions with lower bone mass, such as the femoral neck (FN) and greater trochanter (GT), have gentler slopes of 12.85 and 14.97, respectively.

Figure 6B shows the relationship between the reduction rate of apparent elastic modulus and the reduction rate of bone mass, with the linear relationship slopes decreasing sequentially from the femoral neck to the greater trochanter, central femoral head, upper femoral head, and lower femoral head (with slopes of 1.81, 1.74, 1.68, 1.62, and 1.42, respectively). This indicates that the impact of bone mass reduction on the mechanical performance of bone structure varies across different regions. For example, comparing FH3 and FN, the slopes of the rate of change in elastic modulus with bone mass reduction are 1.42 and 1.81, respectively. Thus, if bone mass decreases by 5%, the apparent elastic modulus of FH3 will decrease by 7.10% (5% × 1.42), whereas the apparent elastic modulus of FN will decrease by 12.05% (5% × 1.81).

The yield strength changed with bone mass similarly to the elastic modulus (Figures 6C, D), but the yield strain exhibited different behavior (Figures 6E, F). Except for the greater trochanter, the apparent yield strain of trabecular bone shows a significant linear relationship with bone mass (R^2^ ≥ 0.97), although the slopes varied widely, resulting in a poor fit to the overall curve (R^2^ = 0.70).

### 3.3 Influence of tissue-level mechanical properties on apparent mechanical properties

**FIGURE 7:**
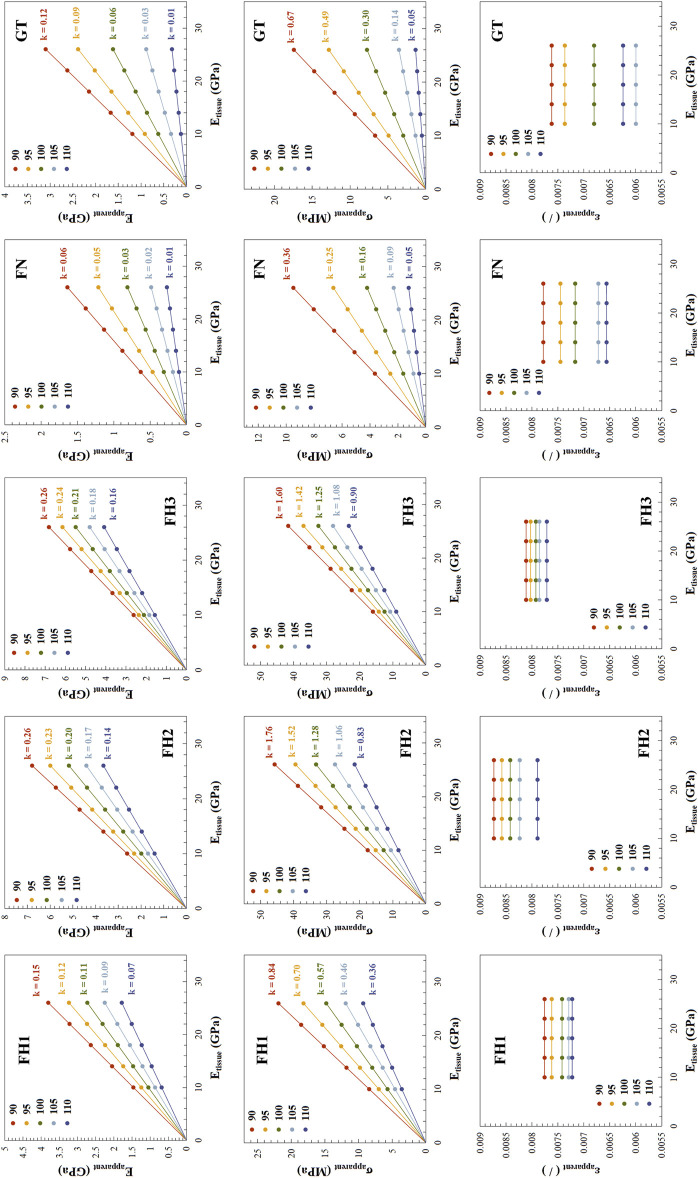
Relationship between apparent mechanical properties and tissue-level elastic modulus across different regions. The structural deterioration under different lower grayscale thresholds is distinguished using various colors. For E_apparent_ and σ_apparent_, the scatter points representing their variation with tissue-level elastic modulus (E_tissue_) lie on lines passing through the origin, with the slope k of these lines indicated in the figure. For ε_apparent_, k is zero, and thus not indicated.

Figure 7 presents the relationship between apparent mechanical properties and trabecular tissue mechanical properties in the femoral neck. The apparent elastic modulus of trabecular structures in different regions of the proximal femur showed a linear relationship with trabecular tissue mechanical properties, with slopes decreasing as structural deterioration progressed. For example, in FH1, as the grayscale threshold increased from 90 to 110, indicating increasing structural deterioration, the slope of the apparent elastic modulus with respect to tissue-level mechanical properties decreased from 0.15 to 0.07. The pattern of yield strength changes was similar to that of the elastic modulus, but yield strain remained unaffected by changes in tissue-level elastic modulus (E_tissue_).

In Figure 7, the linear relationships between apparent elastic modulus and yield strength with tissue-level mechanical properties pass through the origin. This indicates that the ratio of apparent elastic modulus to tissue-level elastic modulus is consistent along the line. As mentioned above, a 5% reduction in bone mass at FH3 and FN can lead to a 7.10% and 12.05% decrease in apparent elastic modulus, respectively, reducing them to 0.929 and 0.8795 times their original values. Based on the proportional relationship, the tissue-level elastic modulus would need to harden to 1/0.929 and 1/0.8795 times their original values, respectively, to restore the apparent elastic modulus to its original level. In this study, the original value of the tissue-level elastic modulus was 18 GPa. Therefore, to offset the negative impact of a 5% reduction in bone mass, the tissue-level elastic modulus at FH3 and FN would need to increase to 19.38 GPa and 20.47 GPa, respectively.

## 4 Discussion

The mechanical properties of trabecular bones are determined by the combined influence of structural features and tissue-level mechanical properties of trabeculae. This study systematically investigates the effects of structural deterioration and material property changes on the apparent mechanical performance of trabecular bone in different regions of the proximal femur. By simulating trabecular bone deterioration and accounting for variations in trabecular material stiffness, we aimed to elucidate the interplay between structural and tissue-level mechanical properties. Our findings reveal that the apparent mechanical performance of trabecular bone generally exhibits a power-law relationship with bone mass. However, uniform deterioration within localized structures results in a linear decrease in apparent mechanical performance. The impact of tissue-level mechanical properties on apparent mechanical performance is determined by the structural characteristics of the trabecular bone.

In this study, we observed a power-law relationship between the apparent mechanical performance and bone mass of trabecular bones from various regions and with varying degrees of deterioration. This distribution aligns with the findings of previous research, which also observed a curved trend in the scatter plots. Typically, the accuracy of simple linear regression models in fitting the mechanical performance of trabecular bone to bone mass and microstructural characteristics is limited, as the data points often follow a curvilinear pattern or are more scattered (Mittra et al., 2005; Smith et al., 2010; Räth et al., 2013; Wang et al., 2015b). Even within the same region, the relationship between mechanical performance and microstructural characteristics across different stages of deterioration usually exhibits complex exponential or power-law curves (Liu et al., 2019; Huang et al., 2023).

However, our study suggests that when focusing on a specific region, uniform structural deterioration leads to a linear decrease in apparent mechanical performance. This may be attributed to the use of a consistent grayscale threshold to simulate the deterioration process, which models uniform structural changes caused by trabecular surface erosion. Despite changes in trabecular structure, the essential structural characteristics remain relatively unchanged from a mechanical transmission perspective. Therefore, the impact of structural deterioration on the apparent mechanical performance of trabecular bone appears linear. This also implies that the actual process of structural deterioration may not be uniform; even within a small region, the deterioration rates of individual trabeculae may vary.

Additionally, we found that the sensitivity of apparent mechanical performance to changes in bone mass varies across different regions. Regions with lower bone mass, such as the femoral neck and greater trochanter, are more significantly affected by the same degree of structural deterioration compared to the femoral head. Previous research indicates that bone mass reduction leads to a substantial decline in mechanical performance, with this decline accelerating as bone mass decreases (Mulvihill et al., 2008; Doolittle et al., 2021; Pignolo et al., 2021), partially supporting our findings. The interaction between structural deterioration and the mechanical environment is reciprocal: structural deterioration alters the mechanical environment, which in turn exacerbates changes in trabecular bone structure (Huiskes et al., 2000; Ozcivici and Judex, 2014; Barak and Black, 2018). Consequently, minor osteoporosis has a greater impact on low bone mass regions like the femoral neck and greater trochanter. When bone loss occurs to the same extent in different regions of the proximal femur due to osteoporosis, this change has a greater impact on the mechanical environment of the femoral neck and greater trochanter. This, in turn, can lead to regional differences in the progression of osteoporosis. The structural characteristics of trabecular bone in different areas of the proximal femur are significantly distinct. Therefore, it is essential to consider these regional differences in the study of osteoporosis progression and in clinical diagnosis. Targeted detection in low bone mass areas may hold significant potential for the early diagnosis of osteoporosis.

Previous studies generally agree that while tissue-level composition or mechanical properties can influence trabecular bone mechanics, their impact is relatively minor compared to microstructural changes (Gong et al., 2016; Liu et al., 2019; Yu et al., 2020; Huang et al., 2023). Our findings partially support this view. The slope of apparent elastic modulus changes with bone mass is much steeper than with tissue-level elastic modulus (Figures 6, 7), indicating a greater impact of structural deterioration on trabecular bone mechanics.

However, our study also highlights the significant role of tissue-level mechanical properties. We found that the apparent elastic modulus of trabecular bone is linearly influenced by tissue-level elastic modulus, with the slope varying according to the inherent structural characteristics. Specifically, higher bone mass in trabecular structures corresponds to higher initial apparent elastic modulus, making changes in tissue-level properties more impactful. Thus, tissue-level mechanical property variations have a greater effect on trabecular bone mechanics in patients with mild osteoporosis compared to those with severe osteoporosis. Our results suggest that in the early stages of osteoporosis, a slight increase in trabecular material stiffness can mitigate the negative impact of bone mass reduction on mechanical performance, which indicates that changes in trabecular material properties could serve as a compensatory strategy against osteoporosis. This aligns with the view that increased trabecular mineralization during osteoporosis may help compensate for bone mass loss (Busse et al., 2009; Brennan et al., 2011; O’Sullivan et al., 2020). Furthermore, our study revealed that the apparent yield strain of trabecular bone is not affected by trabecular material stiffness, indicating that increased stiffness enhances the load-bearing capacity of the trabecular structure within the elastic deformation range. However, this also raises local stress levels. Previous research shows that trabecular mineralization in osteoporosis patients is heterogeneous, and increased heterogeneity in mineralization reduces overall structural strength (Busse et al., 2009; Brennan et al., 2011; Renders et al., 2011). This reduction in strength is likely due to the combined effects of decreased trabecular material toughness and stress concentration. Overall, the decline in the mechanical properties of bone structure caused by bone loss and structural deterioration can be compensated by increasing bone tissue stiffness. Therefore, the mineralization of bone tissue, especially in the early stages of osteoporosis, may not be a negative pathological change but rather a protective strategy of the body. However, as osteoporosis progresses, the significant reduction in bone structural mechanical properties due to severe deterioration cannot be compensated by bone tissue alone. This underscores the necessity for early diagnosis and timely treatment of osteoporosis.

There are several limitations to our study. First, the simulations were based on uniform structural deterioration, which may not fully capture the heterogeneous nature of actual trabecular bone deterioration. Relevant studies have confirmed overall changes during osteoporosis, such as trabecular thinning and morphological transitions to rod-like structures, but the gradual progression of these changes remains unclear. Understanding this progression is crucial for studying the mechanisms of osteoporosis. Detecting osteoporosis in animal models at shorter intervals could enhance this understanding, and non-radiative imaging techniques such as MRI for small animals have significant potential in this field. Second, this study focused solely on the trabecular structure of the proximal femur, so the results may not be directly applicable to other skeletal regions. Third, although our study considered variations in trabecular material stiffness, other factors such as bone remodeling dynamics and biochemical influences were not included.

## 5 Conclusion

Our study provides a comprehensive understanding of how structural and material properties influence trabecular bone mechanics. It is demonstrated that in regions of low bone mass, the mechanical properties of bone structure are particularly sensitive to structural degeneration. This highlights the potential value of regional assessments in the diagnosis of osteoporosis. Additionally, we found that the stiffening of bone tissue can compensate for the negative effects of structural degeneration on bone mechanical properties, especially in the early stages of bone loss. This finding offers a new perspective on the progression of osteoporosis and underscores the necessity for early diagnosis and treatment.

## Data Availability

The raw data supporting the conclusions of this article will be made available by the authors, without undue reservation.
